# All-photonic intercity quantum key distribution

**DOI:** 10.1038/ncomms10171

**Published:** 2015-12-16

**Authors:** Koji Azuma, Kiyoshi Tamaki, William J. Munro

**Affiliations:** 1 NTT Basic Research Laboratories, NTT Corporation, 3-1 Morinosato Wakamiya, Atsugi, Kanagawa 243-0198, Japan

## Abstract

Recent field demonstrations of quantum key distribution (QKD) networks hold promise for unconditionally secure communication. However, owing to loss in optical fibres, the length of point-to-point links is limited to a hundred kilometers, restricting the QKD networks to intracity. A natural way to expand the QKD network in a secure manner is to connect it to another one in a different city with quantum repeaters. But, this solution is overengineered unless such a backbone connection is intercontinental. Here we present a QKD protocol that could supersede even quantum repeaters for connecting QKD networks in different cities below 800 km distant. Nonetheless, in contrast to quantum repeaters, this protocol uses only a single intermediate node with optical devices, requiring neither quantum memories nor quantum error correction. Our all-photonic ‘intercity' QKD protocol bridges large gaps between the conventional intracity QKD networks and the future intercontinental quantum repeaters, conceptually and technologically.

## Abstract (web summary)

Quantum key distribution (QKD) holds promise for unconditionally secure communication, but due to fibre losses distances are so far restricted to intracity. Here, the authors present an all optical QKD protocol that can connect distant cities without the need of quantum repeaters or quantum error correction.

## Graphical abstract

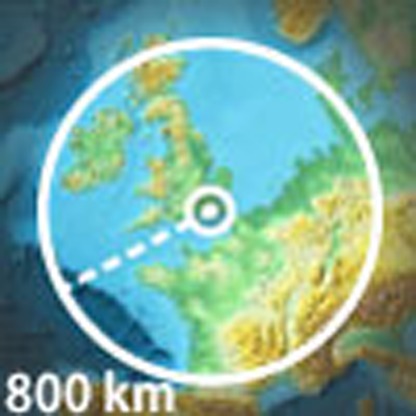

In the conventional world, communication networks are connected to each other with backbone links. This way, a worldwide communication network such as the Internet is formed. Analogously, although recent field demonstrations for intracity quantum key distribution (QKD) networks hold promise for unconditionally secure communication with point-to-point links up to a 100 km (refs 1, 2), such intracity networks will be connected by a backbone quantum link to build a worldwide QKD network in the future. In principle, from its core role, such a backbone quantum link might use more demanding devices than the usual links in the intracity QKD network, for example, in contrast to the cost-effective last-mile service^3^,^4^. Quantum repeaters^5^,^6^,^7^,^8^,^9^,^10^,^11^,^12^,^13^,^14^,^15^,^16^,^17^,^18^,^19^,^20^,^21^,^22^ could be adopted as the backbone quantum link, given that the communication efficiency scales polynomially with the communication distance, compared with the exponential scaling of the conventional QKD links^1^,^2^. This polynomial scaling of quantum repeaters is necessary for intercontinental backbone quantum links. But, otherwise, quantum repeaters are overengineered from the following reasons: Major cities to be equipped with an intracity QKD network may be within a radius <1,000 km, and the polynomial scaling of quantum repeaters usually necessitates quantum memories^5^,^6^,^7^,^8^,^9^,^10^,^11^,^12^,^13^,^14^,^15^,^16^,^17^,^18^,^19^,^20^ or quantum error correction^5^,^7^,^11^,^13^,^17^,^18^,^19^,^20^,^21^—which is extremely challenging as it requires a huge number of qubits as well as many repeater nodes. Therefore, an intercity backbone quantum link—which would be more effective in connecting intracity QKD networks in different major cities than quantum repeaters—may be in greater demand than an intercontinental one based on quantum repeaters, to compose the future worldwide QKD network.

The main point of this paper is to present such an intercity QKD protocol using only a single untrusted intermediate node between communicators. The node uses only single-photon sources, linear optical elements, single-photon detectors, optical switches and active feedforward techniques, requiring neither quantum memories nor quantum error correction, in contrast to other known protocols^5^,^6^,^7^,^8^,^9^,^10^,^11^,^12^,^13^,^14^,^15^,^16^,^17^,^18^,^19^,^20^,^21^,^22^,^23^,^24^. This implies that our protocol also has the following distinct advantages for the implementation. First, the absence of memories implies that the repetition rate can be increased as high as one wants within those allowed by the assumed optical devices. Second, the absence of matter systems makes coherent frequency converters for photons (to strengthen the coupling to matter^25^ and to optical fibres^26^) unnecessary. Finally, our protocol could work at room temperature in principle, thanks to its all-photonic nature. Nonetheless, our scheme leads to a square root improvement in the secret key rate over conventional QKD schemes^1^,^2^,^27^. Moreover, our scheme could supersede even quantum repeater schemes^6^,^10^,^14^ with atomic ensembles for the communication distances below 800 km. From a fundamental viewpoint, our scheme highlights conceptual differences between an entanglement-based QKD scheme^28^,^29^ and its time-reversed version^30^,^31^,^32^—now called^32^ measurement-device-independent QKD (mdiQKD) for the sake of closing all the security loopholes of measurement devices—as well as between QKD protocols and quantum repeaters for providing entanglement.

## Results

### Entanglement-based QKD and mdiQKD

**Figure 1:**
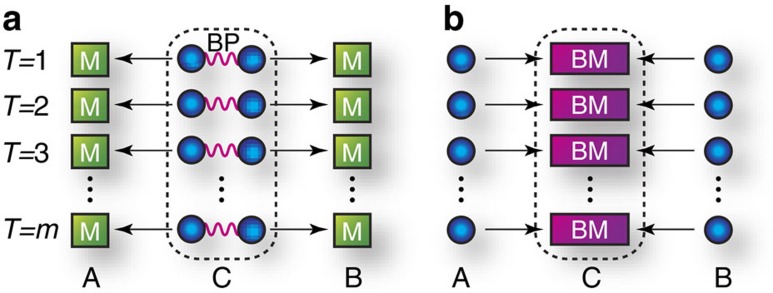
Entanglement-based QKD and mdiQKD. *T* is the trial number. (**a**) In the entanglement-based QKD protocol, node *C* sends halves of Bell pairs (BP) to Alice and Bob who randomly perform *Z*-basis or *X*-basis measurement (M), respectively. (**b**) In the mdiQKD protocol, node *C* performs Bell measurements (BM) on photons that have been prepared randomly in one of the eigenstates of complementary observables 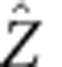 and 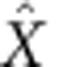 and sent simultaneously by Alice and Bob. These protocols are related by a simple time reversal^32^, requiring 
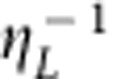
 trials on average to obtain a pair of bits for the secret key.

Our protocol emerges from highlighting a difference between an entanglement-based QKD scheme^28^,^29^ and the mdiQKD scheme^32^. Let us start by considering this. The schemes assume a single untrusted node *C* in the middle of communicators Alice and Bob, separated over distance *L* (Fig. 1). Here node *C* shares optical channels with Alice and Bob, whose transmittance is described by 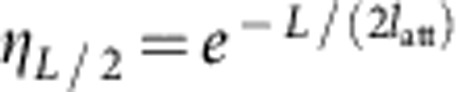 with attenuation length *l*_att_. The transmittance is equal to the arrival probability of a single photon through the lossy channels. Those protocols could provide Alice and Bob with a pair of bits for the secret key only when both photons—exchanged between node *C* and Alice and between node *C* and Bob—survive the loss in the optical channels. Hence, the number of trials required on average to obtain a pair of bits for the secret key is 
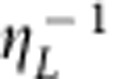
 in both of the protocols. In fact, all known QKD protocols—including prepare-and-measure QKD schemes^1^,^2^ whose final key rates *G* per pulse are now limited^27^ by the Takeoka–Guha–Wilde (TGW) bound 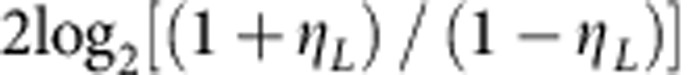 because of the lack of intermediate nodes—share^1^,^2^ this scaling without quantum memories^5^,^6^,^7^,^8^,^9^,^10^,^11^,^12^,^13^,^14^,^15^,^16^,^17^,^18^,^19^,^20^,^23^,^24^ or quantum error correction^5^,^7^,^11^,^13^,^17^,^18^,^19^,^20^,^21^. In contrast, our protocol improves the scaling from 
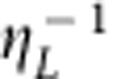
 to 
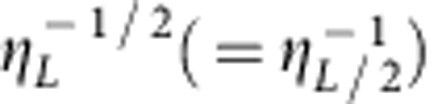
 only with the help of a single node without any of such demanding devices. The essence of our idea is to notice that the original scaling 
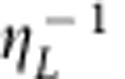
 is caused by a fact that the pairings at node *C* for Bell pairs in the entanglement-based QKD scheme or for Bell measurements in the mdiQKD scheme (cf. Fig. 1) are predetermined independently of the occurrence of photon losses. In other words, to outperform the 
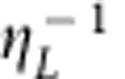
 scaling, we need to make the pairings depend on the occurrences of photon losses. Interestingly, this is possible solely for the mdiQKD protocol, because it entangles photons after the transmission in contrast to the entanglement-based QKD scheme (cf. Fig. 1).

### Basic idea of our adaptive mdiQKD

**Figure 2:**
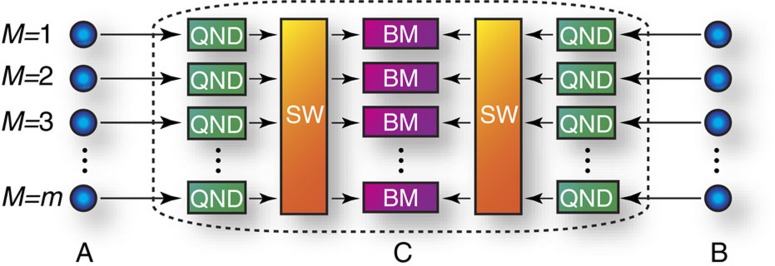
Basic idea of our mdiQKD protocol with an adaptive Bell measurement. *M* is the pulse number. In this protocol, the node *C* first performs quantum non-demolition (QND) measurements to confirm the successful arrival of single photons, followed by optical switches (SW) to send the surviving photons to Bell measurement (BM) modules.

To be precise, we introduce our protocol regarded as an mdiQKD scheme, where node *C* adaptively performs the Bell measurements only on surviving photons under losses (Fig. 2). This protocol proceeds as follows: (i) Alice and Bob send *m* optical pulses in single-photon states—each of which is randomly selected from the eigenstates of complementary observables 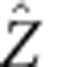 and 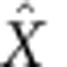
—to node *C* simultaneously, using multiplexing. (ii) On receiving the pulses, node *C* applies quantum non-demolition (QND) measurements to the pulses to confirm the arrival of the single photons over lossy channels. (iii) Then, successfully arriving photons from Alice are paired with ones from Bob via optical switches at node *C*. (iv) Node *C* then performs a Bell measurement on each of these pairs. (v) Node *C* then announces the pairings and the measurement outcomes of the Bell measurements. (vi) Finally, as bits for the secret key, Alice and Bob keep the eigenvalues corresponding to their sent eigenstates to which the Bell measurements have been successfully applied. The bits obtained in step (vi) will be processed with a manner similar to the data that are kept after the quantum communication phase of the original mdiQKD protocol^32^.

Let us consider the scaling of our protocol. When Alice's and Bob's pulses are perfectly in single-photon states, the transmittance *η*_*L*/2_ of the channels affects only the probability of confirming the arrival of single photons via QND measurements in step (ii). Since this probability is proportional to, if the number *m* of multiplexing is larger than 
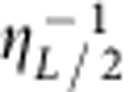
, one or more single photons arrive at node *C* from each of Alice and Bob with a high probability. Since the successful application of the Bell measurement to these single photons leads to a pair of bits for the secret key in step (vi), the communication resources such as required optical pulses and devices—which are proportional to the number *m* of the multiplexing—are in the order of 
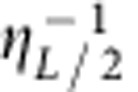
. This is a square root improvement over conventional protocols^1^,^2^, which results from making the pairings for the Bell measurement depend on the successful arrival of single photons.

More precisely, our protocol has a direct impact on the asymptotic sifted-key generation rate 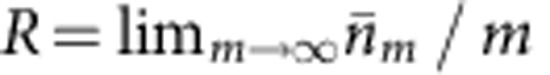, where 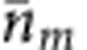 is the average number of the sifted pairs for the number *m* of multiplexing. *R* is included in the final key rate formula *G* per pulse (normalized by the number of events of the same basis choice by Alice and Bob) as^1^


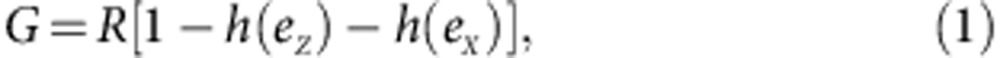


where *h*(*x*) is the binary entropy function defined by 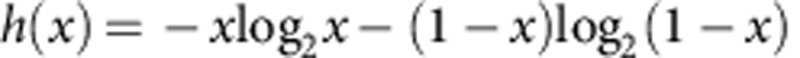 and *e*_*Z*_ (*e*_*X*_) is the error rate for Alice's and Bob's choice of *Z*-basis (*X*-basis)—called the bit-error rate (the phase-error rate). *R* for our protocol is given by


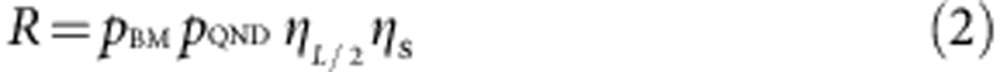


for Alice's and Bob's photon sources with efficiency *η*_s_, QND measurements with success probability *p*_QND_ and Bell measurements with success probability *p*_BM_ (see Methods). As the rate of the original mdiQKD protocol is 
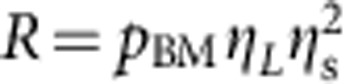
, our protocol necessitates, at least,


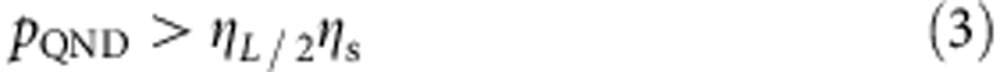


to outperform it in terms of *R*. Given that *p*_BM_ contributes to 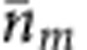 independently of *m* (see Methods), the number of multiplexing should be 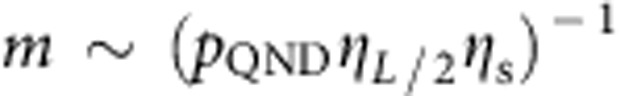 to obtain *R* in the order of equation (2).

### All-photonic implementation

To implement our protocol, we only need optical devices. The Bell measurement in step (iv) can be conducted just by using linear optical elements and single-photon detectors^33^, similarly to the original mdiQKD scheme^32^. A challenging technique in our protocol is the QND measurement in step (ii). Besides many schemes for the QND measurement involving matter qubits or matter quantum memories, fortunately, there are several all-photonic schemes for the QND measurement for single photons^33^. Here we focus on a simple example, that is, a QND measurement for a single photon^34^ based on quantum teleportation^35^. This scheme teleports the single-photon state of the incoming pulse to that of a half of a photonic Bell pair via the linear-optics-based Bell measurement, using the feature that the teleportation fails when the incoming pulse is in the vacuum state.

The protocol composed of steps (i)–(vi) is now implementable by using optical devices alone. However, the optical switch required in step (iii) may still be challenging because it should have the input modes in the order of 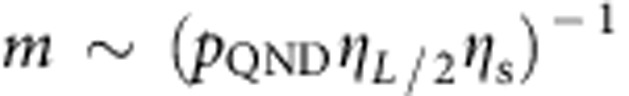 (for one or a few output modes). In particular, a large-scale optical switch to route a single photon in one of the many input modes into a Bell measurement module in step (iv) may be much more difficult than the existing ones^36^,^37^,^38^ with a small number of input modes. For instance, although we can realize an *m* × 1 optical switch by concatenating 2 × 1 optical switches with transmittance *η*_sw_ in a knockout tournament manner with depth 
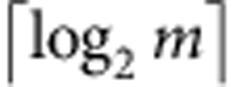
, the transmittance of the large-scale optical switch decreases as 
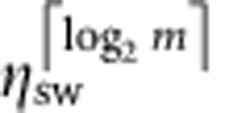
, which may thus be needed to be taken care of in this case. However, remarkably, it is also possible to perform our protocol without using such a large-scale optical switch, that is, by using only single-mode on/off switches, a passive Hadamard linear optical circuit and single-photon detectors.

To achieve our protocol without large-scale optical switches, steps (iii)–(v) can be replaced with the followings: (iii') Then, a mode *i* (*i*=1, 2, …, *m*) with a successfully arriving photon from Alice and a mode *j* (*j*=*m*+1, *m*+2, …, 2*m*) with a successfully arriving photon from Bob are directly sent to the Hadamard linear optical circuit that acts on the 2*m* modes of node *C* as 
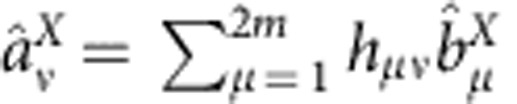
 with an orthogonal 2*m* × 2*m* Hadamard matrix *H*=[*h*_*μν*_] and annihilation operators 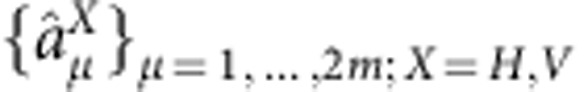 
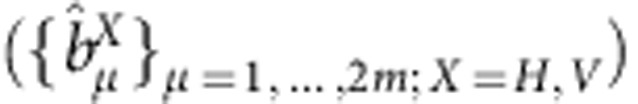
 for the input (output) modes and their orthogonal polarizations *H* and *V*. Except for two modes *i* and *j*, all the optical modes are blocked off with the single-mode on/off switches. (iv') Node *C* then measures all the 2*m* output modes of the Hadamard linear optical circuit with polarization discriminating photon counters, and, if a photon with polarization *H* is found in output mode *k* and a photon with polarization *V* is found in output mode *l* (*k*, *l*=1, 2, …, 2*m*), it regards this trial as successful application of a Bell measurement showing that input modes *i* and *j* have been in unnormalized Bell state 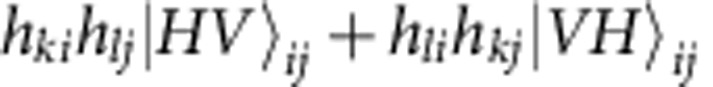. (v') Node *C* then announces input modes *i* and *j* and output modes *k* and *l*.

In the modified protocol here, since the sifted-key generation rate *R*=*P*_*m*_/*m* with success probability *P*_*m*_ of the protocol and error rates *e*_*X*_ and *e*_*Z*_ in the formula for the final key rate *G* per pulse are the functions of the number *m* of multiplexing, *m* should be chosen to maximize *G*, but 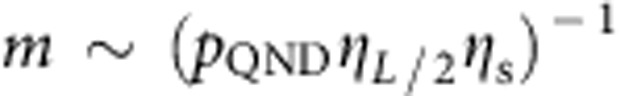 gives the maximum of *G*. The property of the Hadamard matrix that all the elements are 
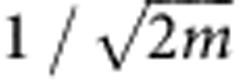
 or 
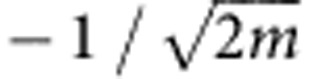
 would be needed to suit the phase-error estimation in the mdiQKD (ref. 32). In fact, thanks to this property, the sequence of (iii')–(v') essentially performs a Bell measurement to distinguish Bell states 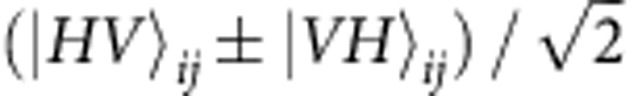 from the other states, and the phase-error estimation in the original mdiQKD protocol^32^ thus works even for our modified mdiQKD scheme in the same way. However, the Hadamard matrix exists only on restricted dimensional vector spaces, in contrast to a general Fourier transformation. For instance, it exists on 2^*s*^-dimensional vector spaces with s=1, 2, …. Hence, we use the Hadamard matrix on 2^*s*^-dimensional vector spaces with 2^*s*^=2*m*, based on Sylvester's construction. The symmetry of this construction is indeed favourable for calculating the performance of the modified protocol, because the effects of non-unity quantum efficiency of single-photon detectors in step (iv') can be regarded as losses in the input modes of the Hadamard linear optical circuit.

### Performance of our all-photonic scheme

We now estimate the final key rate *G* for the original protocol with (iii)–(v) and the modified one with (iii')–(v'), assuming the all-photonic QND measurement based on quantum teleportation for step (ii). Our protocol needs an active feedforward technique with an optical switch. Suppose that a single active feedforward can be completed within time *τ*_a_, during which photons run in optical fibres, being subject to the corresponding loss. In addition, we assume single-photon sources with efficiency *η*_s_ that emit pulses with duration *τ*_s_ and single-photon detectors with quantum efficiency *η*_d_ and with dark count rate *ν*_d_. For simplicity, despite the being of various schemes for single-photon sources^39^, since our protocol, in any case, necessitates the active feedforward technique, we assume a single-photon source^36^,^37^,^40^ based on multiplexing of heralded single-photon sources. In fact, this photon source holds^40^ promise for producing high-fidelity telecom single photons with the repetition rate of the slowest optical device at the expense of the use of (at least) one active feedforward, and it would be realizable just by using only a small amount of multiplexing^41^,^42^. Bell pairs for the all-photonic QND measurements in step (ii) can be generated in constant time *τ*_a_ with single-photon sources rather than a Bell-pair photon source, by paralleling a probabilistic procedure^43^ with the active feedforward technique. In practice, this kind of step-wise preparation of Bell pairs may be useful for suppressing the unnecessary multi-photon components, because such multi-photon components may just contribute to events to be discarded as failure (as this kind of phenomenon indeed occurs sometimes^44^). In addition, note that we need to use one active feedforward in step (iii) or (iii').

**Figure 3:**
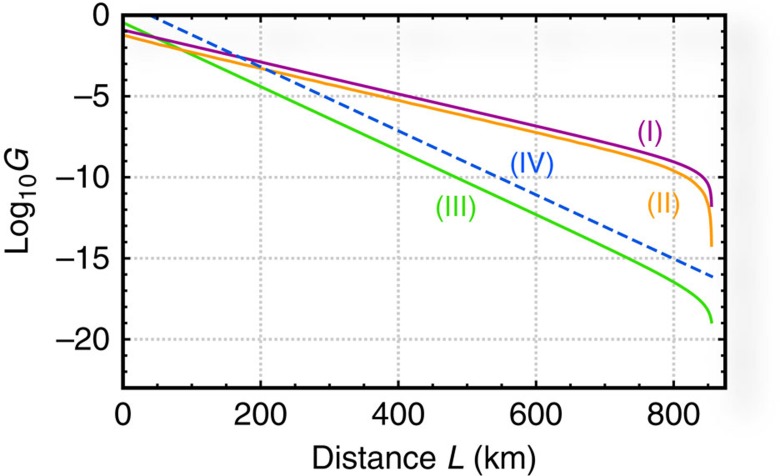
Secret key rates *G* per pulse versus distances *L*. *G* is normalized by the number of events of the same basis choice by Alice and Bob. Here *η*_s_=0.90 (refs 40, 45, 46), *τ*_s_=100 ps (ref. 47), *η*_d_=0.93 (ref. 48), *ν*_d_=1 s^−1^ (refs 48, 49), *τ*_a_=67 ns (ref. 36), *l*_att_=22 km and *c*=2.0 × 10^8^ m s^−1^. Lines (I)–(IV) represent our original protocol with steps (iii)–(v), our modified protocol with steps (iii')–(v'), the original mdiQKD protocol^32^ with the same single-photon sources and the TGW bound^27^ 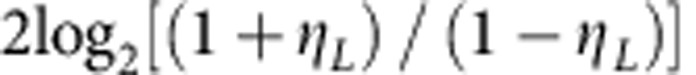, respectively.

Under these assumptions, the final key rates *G* are illustrated in Fig. 3 by assuming a collection of the state-of-art technologies^36^,^40^,^45^,^46^,^47^,^48^,^49^. Although the modified protocol merely uses the Hadamard matrix on 2^*s*^-dimensional vector spaces with 2^*s*^=2*m*, the key rates *G* labelled line (II) in Fig. 3 look like continuous for distance *L*, implying that the restricted choice of the Hadamard matrices is not a problem. Figure 3 shows that both of our original and modified protocols outperform the original mdiQKD protocol^32^ (the TGW bound^27^) for distances *L* larger than ∼100 km (∼200 km). These crossing distances are much smaller than those for quantum repeaters (for example, ∼500 km for protocols^14^ based on atomic ensembles). Moreover, the performance of both our protocols is seven orders of magnitude better than that of the original mdiQKD protocol for *L*=800 km. Since the assumed state-of-art technologies^36^,^40^,^45^,^46^,^47^,^48^,^49^—including the synchronization as seen in the experimental demonstrations^50^,^51^,^52^,^53^,^54^,^55^ of the original mdiQKD (ref. 32)—work with 15 MHz at least^36^, the key generation rate per second of our original protocol (the modified one) is then 1.7 kHz (0.69 kHz) for *L*=307 km, which is a couple orders of magnitude better than experimental demonstrations^47^,^56^ of QKD over the current record distance. More interestingly, the rate is 13 mHz (3.8 mHz) for *L*=800 km, which is the same order of (only one order of magnitude less than) that of the best quantum repeater scheme^10^ with atomic ensembles^14^. It is then clear that both of our schemes outperform the best quantum repeater scheme^10^ below 800 km, if all the optical components work with 1 GHz as predicted to be possible^14^,^36^,^53^,^54^. The cutoff distances of 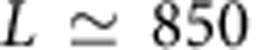 km for both protocols in Fig. 3 are determined by the signal-to-noise ratio associated with the dark counting of the single-photon detectors. But the cutoff distances could be extended^57^ if we replace the prepare-and-measure scheme of Fig. 2 between Alice (Bob) and node *C* with an entanglement-based one by putting an additional node with Bell-pair sources in between them.

## Discussion

We have presented an adaptive mdiQKD scheme that can present a square root improvement over conventional QKD schemes^1^,^2^,^27^, superseding even quantum repeaters^14^ for intercity distances. The ‘adaptive' Bell measurement performed by node *C* in our scheme is also useful for providing a square root improvement for any single-photon-based entanglement generation protocol, for example, entanglement generation schemes for quantum repeaters with atomic ensembles^14^. However, note that it is impossible for our protocol alone to serve as quantum repeaters blessing an exponential improvement. In fact, although we can use our protocols as the entanglement generation for Alice's and Bob's stationary qubits by starting from entangling their photons with their stationary qubits, they need to wait the arrival of the heralding signals from node *C* in step (v) or (v') to identify the stationary qubits that have successfully been entangled, which is impossible without the memory function of their stationary qubits. This is an unbridgeable gap between our QKD protocol and quantum repeaters, and hence, for extremely long distances such as thousands of kilometres, quantum repeaters are needed. However, combined with all-photonic quantum repeaters^21^, our protocol certainly paves a seamless route towards the all-optical realization of a worldwide QKD network—which would be not only a certain milestone^21^ towards the all-photonic quantum computation^43^,^58^ but also an ultimate challenge for the all-optical approach^59^ in the field of conventional communication. Our protocol would also lead to unforeseeable attractive new twists—such as the realization of telescope arrays with much longer baselines than existing facilities^60^ without quantum repeaters, the understanding of the fundamental limit for intercity/intercontinental quantum communication beyond the TGW bound and the finding of more practical variants of our protocol (for example, based on the combination of the time multiplexing with ultrafast single-photon sources for reducing the number of the QND measurement modules and on the hybridization of moderate-size optical switches and Hadamard-circuit-based Bell measurements for decreasing the number of the required single-photon detectors).

## Methods

### Asymptotic sifted-key generation rate

The asymptotic sifted-key generation rate *R* of our protocol can be evaluated as follows. The probability *p*_*k*|*m*_ with which node *C* finds the existence of *k*(≤*m*) single photons from Alice or Bob via QND measurements in step (ii) is


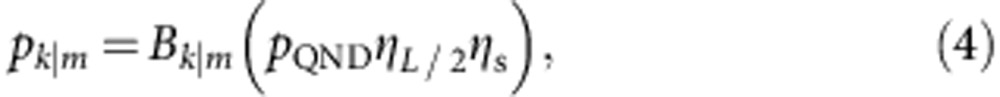


where *B*_*k*|*m*_(*p*) is the binomial distribution with 
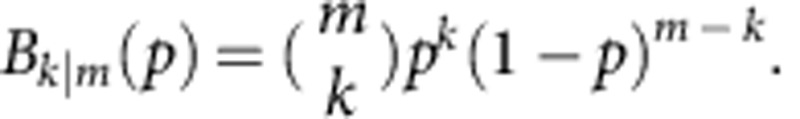
 To make *l* pairs in step (iii), the node *C* should have found the existence of single photons ≥*l* from both of Alice and Bob in step (ii), which occurs with probability 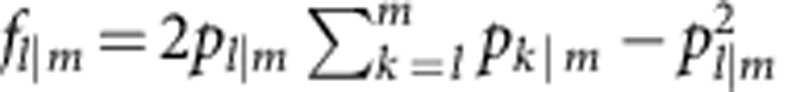. Hence, the probability 
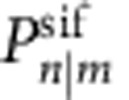
 with which our protocol provides *n* pairs of bits for the sifted key in step (vi) is described as


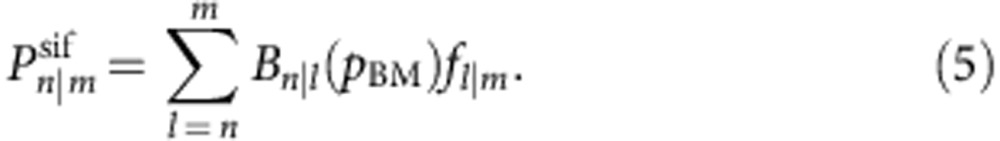


The average number 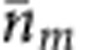 of sifted pairs is then


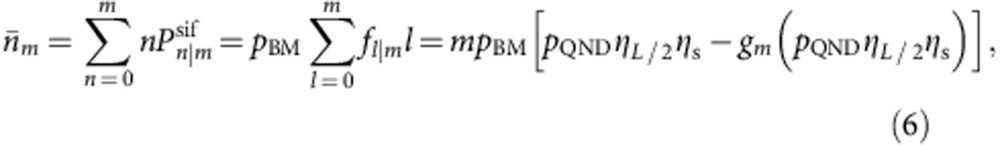


where *g*_*m*_ is shown to be


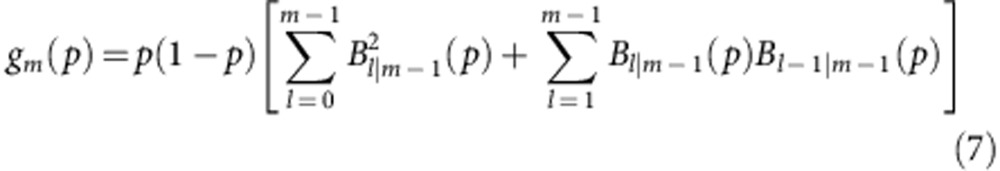


by using *lB*_*l*|*m*_(*p*)=*mpB*_*l*−1|*m*−1_(*p*) for *l*>0 and *B*_*k*|*m*_(*p*)=(1−*p*)*B*_*k*|*m*−1_(*p*)+*pB*_*k*−1|*m*−1_(*p*) for 0<*k*<*m*. Since the maximum of *B*_*l*|*m*−1_(*p*) over *l* goes to zero in the limit of *m*→∞, we have lim_*m*→∞_ *g*_*m*_=0. Therefore, the asymptotic sifted-key generation rate 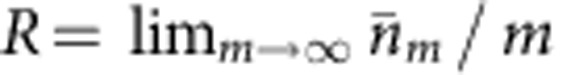 is described by equation (2).

## Additional information

**How to cite this article:** Azuma, K. *et al*. All-photonic intercity quantum key distribution. *Nat. Commun.* 6:10171 doi: 10.1038/ncomms10171 (2015).
